# Using Facebook to promote the uptake of colorectal cancer screening

**DOI:** 10.1186/s12889-022-12732-w

**Published:** 2022-02-15

**Authors:** Arlinda Ruco, Nancy N. Baxter, Jenna Jacobson, Jill Tinmouth, Diego Llovet

**Affiliations:** 1 Li Ka Shing Knowledge Institute, St. Michael’s Hospital, Unity Health Toronto, Toronto, Canada; 2 Institute of Health Policy, Management and Evaluation, University of Toronto, Toronto, Canada; 3 Melbourne School of Population and Global Health, University of Melbourne, Melbourne, Australia; 4 Ted Rogers School of Management, Ryerson University, Toronto, Canada; 5 Clinical Institutes and Quality Programs, Ontario Health (Cancer Care Ontario), Toronto, Canada; 6 Department of Medicine, Sunnybrook Health Sciences Centre, Toronto, Canada

**Keywords:** Social media, Cancer screening, Mass screening, Facebook

## Abstract

**Background:**

The use of social media presents a unique opportunity for cancer screening programs to motivate individuals to get screened. However, we need a better understanding of what types of social media messages for colorectal cancer (CRC) screening are preferred. The objective of this study was to develop social media messages promoting CRC screening uptake to identify messages preferred by the target audience.

**Methods:**

We conducted a qualitative descriptive study and collected data through focus groups with Facebook users of screen-eligible age. Participants were presented with social media messages and asked to provide feedback. Messages were informed by the Health Belief Model, current evidence regarding screening communication and health communication and social media best practices. Focus groups were audio-recorded and transcribed and analysis was completed by two independent coders. If messages generated sufficient discussion, we developed a recommendation regarding the use of the message in a future social media campaign. Recommendations included: strongly consider using this message, consider using this message, proceed with caution, and do not use this message. General considerations about social media campaigns were also noted.

**Results:**

A total of 45 individuals participated in six focus groups. We developed recommendations for 7 out of the 18 messages tested; 1 was classified as strongly consider using this message, 4 as consider using this message and 2 as proceed with caution. The data suggest that participants preferred social media messages that were believed to be credible, educational, and with a positive or reassuring tone. Preferred messages tended to increase awareness about CRC risk and screening and prompted participants to ask questions, and to want to learn more about what they could do to lower their risk. Messages that were viewed as humorous, strange or offensive or that had a negative or excessively fearful tone were less well received by study participants.

**Conclusions:**

Facebook users prefer social media messages for CRC that have a positive or reassuring tone, are educational, and that have a credible ad sponsor. Campaign planners should proceed with caution when considering messages that use humor or a fearful tone to avoid undermining their campaign objectives.

**Supplementary Information:**

The online version contains supplementary material available at 10.1186/s12889-022-12732-w.

## Background

Over 19 million new cancer cases were diagnosed globally in 2020 with approximately 10 million cancer deaths [1]. Colorectal cancer (CRC) is one of the most common cancers, surpassed only by breast and lung cancer [1]. Approximately 10% of all new cancer cases and 9.4% of cancer deaths can be attributed to CRC alone [1]. One strategy to reduce incidence and mortality of cancer is through screening. Screening has been shown to improve cancer outcomes through early detection and prevention of the disease [2, 3]. Organized screening programs are able to define a target population appropriate for screening, send invitations, reminders, and recalls, and track outcomes [4]. However, in many jurisdictions, including those with and without an organized screening program, participation in CRC screening remains suboptimal [5]. For example, an overview of screening programs across the world reported that participation rates for cancer screening with stool-based testing range from 16 to 77% in the first round [5]. This suggests, that a large proportion of eligible individuals are not getting screened. Different and innovative approaches are needed to maximize screening participation in order to reduce the public health burden of the disease.

While traditional mass media campaigns can be used to reach a large proportion of the target population through popular media outlets (e.g. television, radio and print media), these are generally costly and the prominence of such outlets is also declining [6]. Social media is increasingly being used for health promotion and behaviour change interventions, [7–9] and affords users the ability to create, discuss and share content in online communities or networks. Individuals use these platforms to access information and connect with others through frequent interactions such as posts or stories that may include tweets, photos, or videos depending on the platform. Social media is now being utilized as a major communication tool across all sectors including healthcare [10]. The bidirectional flow of information, the potential for anonymity in interactions, and the ability to reach and engage individuals from across the globe with relatively low cost makes social media attractive to many users [10].

Evidence has also started to emerge regarding the specific use of social media for cancer screening, prevention and management [7, 10–17]. Literature on the use of theory-based social media interventions for promoting cancer screening is limited, with a limited number of studies focused specifically on CRC [17]. A better understanding of what types of social media messages for CRC screening participation specifically are preferred is needed. Facebook is the most popular social media platform among the population that is eligible for CRC screening (50–74 years). It is estimated that approximately 76% of those aged 55+ use Facebook in comparison with other platforms like Instagram (28%), LinkedIn (40%), Twitter (27%), Pinterest (30%), or Snapchat (6%), [18, 19] making Facebook the most appropriate platform for our study.

The objective of this study was to develop social media messages promoting CRC screening uptake on Facebook with users of screen-eligible age (50–74) and to identify messages preferred by the target audience.

## Methods

We developed social media messages promoting CRC screening and evaluated them using a qualitative descriptive study. The study was performed in accordance with relevant guidelines and regulations and informed consent was obtained from all participants. The study was approved by the Research Ethics Board at St. Michael’s Hospital, Unity Health Toronto (REB# 19–084). We used the Standards for Reporting Qualitative Research (SRQR) checklist to report on our findings [20].

### Social media message specifications

**Fig. 1:**
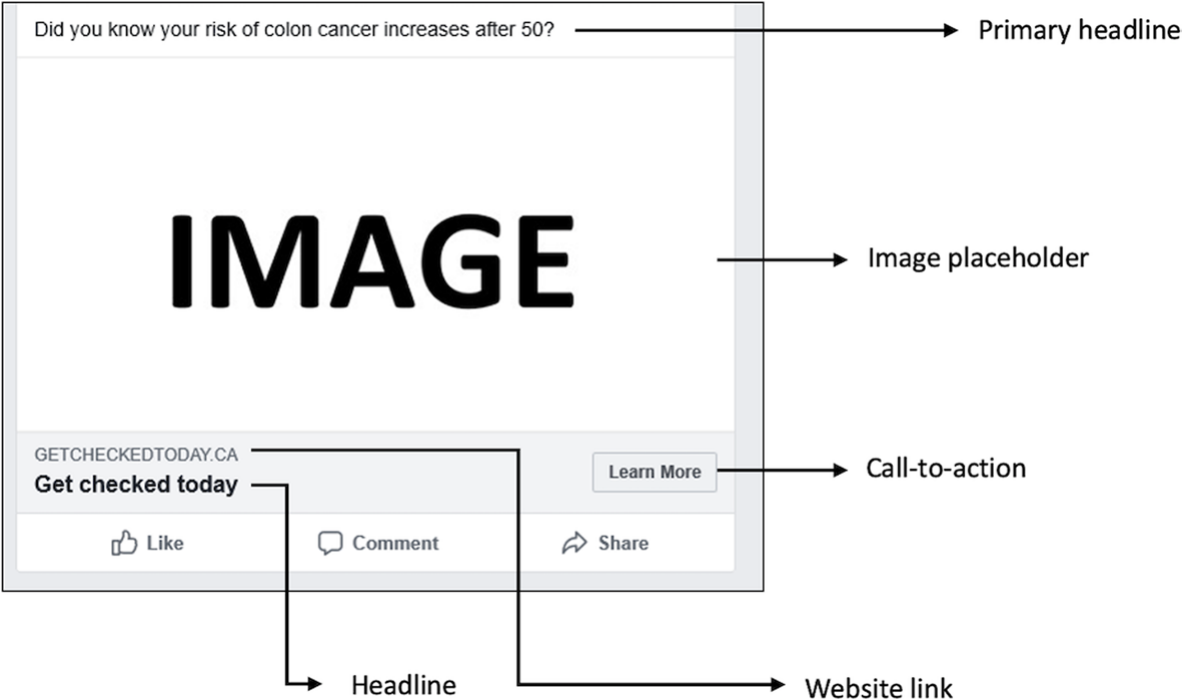
Example of a message as shown to focus group participants with the Facebook ad components outlined

Facebook ads can include an image, primary text, a headline, a description and a call-to-action (Fig. 1) [21]. The primary text appears at the top of the post, and should be 125 characters in length or less as per Facebook recommendations [21]. The headline usually appears right below the image and is recommended it not be longer than 40 characters. The description (30 characters) appears below the headline and can include a website link where users are redirected to if they click on the ad. The call-to-action button invites users to engage with the post. For example, a common call-to-action for Facebook posts includes *‘Learn More’* where users are invited to find out more information about the post.

### Development of social media messages

**Fig. 2:**
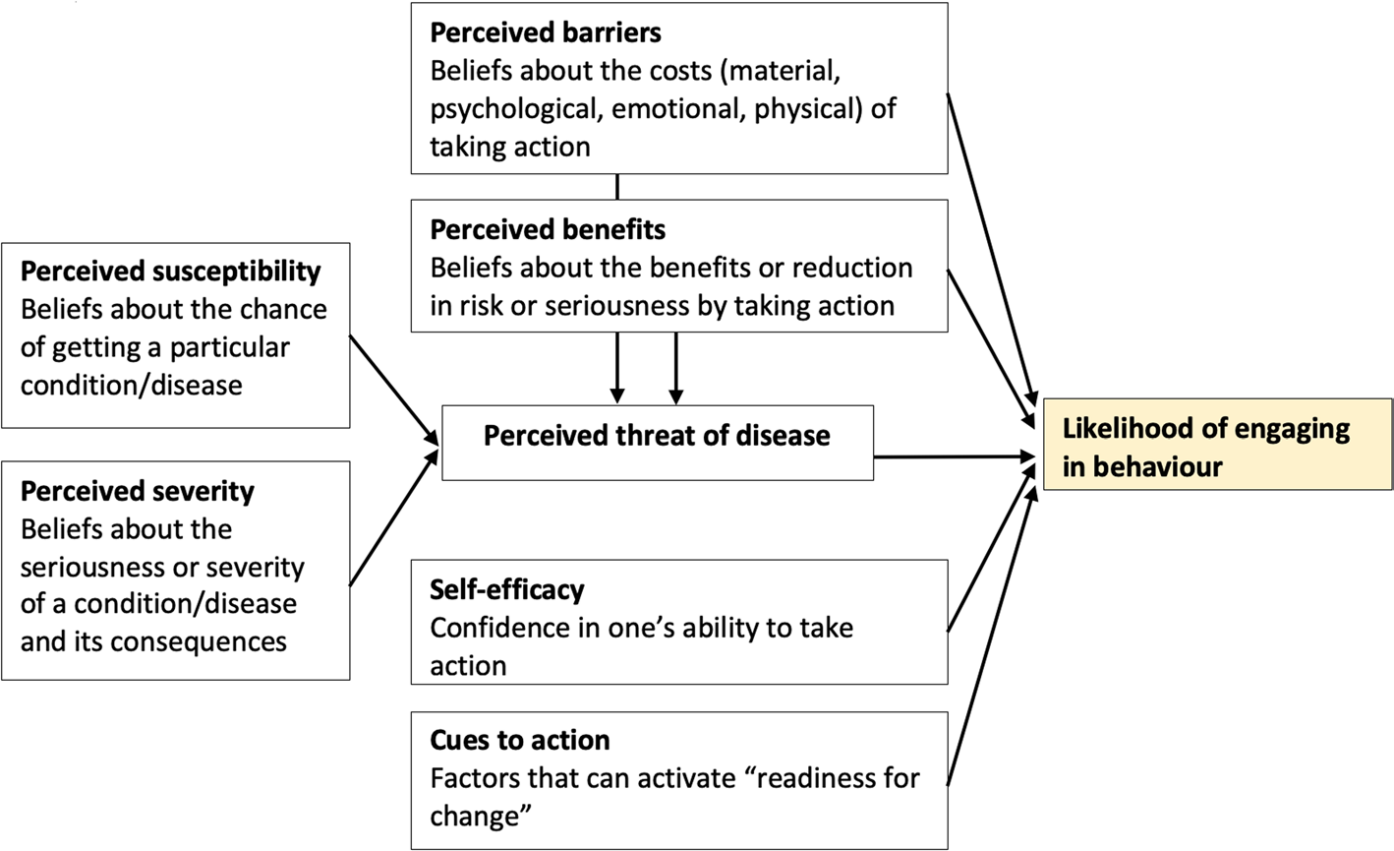
Health Belief Model constructs and definitions [21].

We developed 18 messages (Additional File 1) informed by the Health Belief Model (Fig. 2), [22, 23] current evidence regarding screening communication, [24, 25] messages used in other jurisdictions and campaigns, expert consultations (staff at Ontario Health (Cancer Care Ontario) who have experience developing screening communications) and health communication best practices [23, 24]. The Health Belief Model was selected because a systematic review on individual-level factors for behaviour change pertaining to CRC screening found the most supportive evidence for this behaviour change theory [22]. Messages were written in plain language using available health plain language thesauri [26, 27]. An iterative process with several rounds of feedback was carried out to refine messages. Additionally, a marketing firm executive and copy editor with experience in social media campaigns in the healthcare industry reviewed the messages and revised them according to best practices. We also ensured we had variability among the messages regarding tone, framing, vividness, emotion and source of the message. Each message was classified according to the construct in the Health Belief Model.

### Participants and recruitment

Participants included screen-eligible age individuals (50–74 years) who were current Facebook users (had an active user account), resided in Ontario and could communicate in English. An external research firm was contracted to conduct recruitment of participants using random digit dialing (inclusive of landlines and cell phone numbers) between February and April 2020. For focus groups conducted virtually, participants were also required to have access to appropriate technology to be able to join an online discussion. We aimed to recruit participants for six focus group as previous work empirically assessing saturation within focus groups found that five focus groups was enough for saturation [28].

### Data collection

We collected data through focus groups held in person in the Greater Toronto Area or virtually (due to the COVID-19 pandemic) with residents in Southwestern, Eastern, and Northern Ontario to ensure geographic variation in our sample. Participant socio-demographic information was collected using a questionnaire. A facilitator led all focus groups using an interview guide with prompts to generate discussion and obtain feedback on the messages. Messages were shown in the same format as they would appear in a Facebook post with an image placeholder (Fig. 1). Due to the time limit, it was not feasible to test all messages in every focus group. Each focus group lasted 90 min and participants received a $110 honorarium. The research team included both experienced and early career researchers in the field of cancer screening and prevention, behavioral science, and consumer and producer perspectives on social media. The research team had no prior relationship with focus group participants.

### Data analysis

Focus group discussions were audio-recorded, transcribed, and reviewed for quality assurance prior to analysis. NVivo 12 software (QSR International) was used for data management. The focus group transcripts were analyzed by two independent coders (AR, DL). The coders met multiple times to review the data; they resolved any discrepancies through discussion. Participant comments were identified as relevant if they directly addressed any of the messages being tested. Relevant participant comments were classified as positive or negative (magnitude coding) depending on the sentiment expressed, and were summarized using short phrases that mirrored the language used by the participant (in vivo coding) [29]. The coders then reviewed the data for all messages and selected messages for further analysis where sufficient discussion in focus groups and a range of opinions was received in order to support a confident recommendation regarding the use of the message.

Selected messages were assessed to determine the extent to which they might support the uptake of timely and appropriate stool testing for CRC screening. For this assessment, we considered the participant comments against a list of outcomes that are known to precede the adoption of a health behaviour [30]. For example, ability to recall the message, beliefs about its relevance, and reported knowledge gains from the message can contribute to individual behaviour change [30]. We also considered the severity of the negative comments to distinguish messages that might be only ineffective from those that might be harmful or those with a mixed reaction. The balance between positive and negative comments was considered to offer a recommendation regarding the use of each of the selected messages in a future social media campaign. Initial recommendations were made by the two coders independently and the results were discussed with the broader study team to finalize the recommendations. Recommendations included: strongly consider using this message, consider using this message, proceed with caution, and do not use this message. Comments that provided general insight into how to design a social media campaign for CRC screening were also identified and summarized. Participant socio-demographic characteristics were reported in descriptive summary form.

## Results

A total of 45 individuals participated in six focus groups (Table 1). Briefly, just over half of the participants were female (56%, *n* = 25) and most participants were between the ages of 55–64 (58%, *n* = 26). Twenty-one participants (47%) had previously had CRC screening with colonoscopy being the most common type of screening test completed. Over 70% of participants reported using Facebook at least 2–3 times/week (*n* = 32) and 98% (*n* = 44) reported using Facebook at least once a week.

**Table 1:** Demographic information of focus group participants

	N (%)
**Sex**	
Male	20 (44.4)
Female	25 (55.6)
**Age**	
50–54	9 (20.0)
55–59	13 (28.9)
60–64	13 (28.9)
65–69	7 (15.6)
70–74	3 (6.6)
**Highest level of education completed**	
High school	25 (55.6)
College/University	18 (40)
Graduate school	2 (4.4)
**Annual household income before taxes**	
< $25,000	9 (20)
$25,000–<$50,000	13 (28.9)
$50,000–<$100,000	15 (33.3)
***≥*** $100,000	7 (15.6)
**Employment status**	
Full-time	16 (35.6)
Part-time	10 (22.2)
Unemployed	4 (8.9)
Retired	15 (33.3)
**Ever screened for CRC**	
Yes	21 (46.7)
No	24 (53.3)
**Screening test(s) completed if ever screened for CRC**	
Colonoscopy	16 (76.2)
Stool-test	9 (42.9)
Flexible sigmoidoscopy	2 (9.5)
Colonography	1 (4.8)
**Facebook use frequency**	
Daily	21 (46.7)
2–3 times/week	11 (24.4)
Once a week	12 (26.7)
2–3 times/month	1 (2.2)

We developed recommendations for 7 out of the 18 messages tested in focus groups (Table 2). Briefly, only one message (#4) addressing perceived barriers to getting screened received a recommendation to strongly consider using the message. Participant comments suggested that the message may be effective because it is credible, educational, comforting, and positive. Participants suggested the message addressed barriers to screening by clarifying that it is possible to get screened without undergoing a rectal test or a colonoscopy. No evidence that the message may be ineffective or harmful was found.

**Table 2:** Messages with an overall recommendation along with a summary of the rationale and sample quotes

Message (Behavioural construct)	Recommendation / Notes about sociodemographic characteristics of focus groups whose participants commented on this message	Summary of rationale and sample quotes
#1 – Did you know your risk of colon cancer increases after 50? (Perceived susceptibility)	Consider using this message (with revisions) • *N* = 24; 10 Men and 14 Women across 3 focus groups • Wide range of ages but skewing younger • Ethnically diverse including Caucasian, Asian, Black and West Indian • Range of educational attainment but skewing more highly educated	• **Evidence that the message may be effective** -Message prompted participants to ask questions about the risk of colon cancer *“You have a higher chance when you get 50 years old. So, you only get this when you [turn 50]?” MP9, FG3* -Participants agreed that getting screened is a good idea *“[This message] is good. It’s better to get tested.” MP6, FG3* -Message increased awareness about CRC risk and would encourage participants to seek more information (by clicking on the message) about how to lower their risk *“[This message] was thought provoking. I didn’t really think about the fact that [the risk of] this terrible disease might increase over the age of 50. I probably would click on it to try to find out why would that be, and what might I do to prevent my own risk.” FP7, FG2* -Message is credible *“Yes, [I knew my risk went up at age 50]. When I turned 50, the kit came right to my home. And I did it immediately and every two years now.” MP5, FG1* • **Evidence that the message may be ineffective but not harmful** -Message is bland and does not stand out *“People are consumed with so many things in their lives like global warming, the economy. This statement is sort of bland. It’s like ‘yeah, I know, I know, I’ll get to it’ sort of attitude about it.” FP4, FG1* -Message is not scary enough to motivate someone to act *“I just don’t find this strong enough. In some cases, people need to be scared to do something.” MP3, FG1* -Message does not say if risk of getting colon cancer is high enough to warrant taking preventative action *“What is the incremental amount that it increases after 50? Should I get worried yet?” FP14, FG2* • **Evidence that the message may be harmful** -Participants saw the message as being inaccurate because you can have cancer before age 50 *“Doctors should recommend [screening] when you reach 40. Is 50 too late? Like when you find out you’re at Stage 4.” FP2, FG1* • **Recommendations to improve the message** -Reinforce the magnitude of CRC risk increase after age 50 to make the message more persuasive
#2 – You could have colon cancer right now – and have no clue. (Perceived susceptibility)	Consider using this message • *N* = 45; 20 Men and 25 Women across all 6 focus groups • Wide range of ages • Ethnically diverse including Caucasian, Black, Asian and West Indian • Range of educational attainment	• **Evidence that the message may be effective** -Message is credible *“What I like about it is it’s telling the truth. Some people don’t have symptoms and by the time you go to the doctor because it exhibited some kind of symptoms and by the time they get tested, it’s Stage 4.” FP13, FG2* -Message tone is direct and to the point “*I really like [this message]. It’s direct and to the point.” FP24, FG6* -Message promotes awareness of CRC risk *“I like that, the not knowing. That resonates with me.” MP20, FG6* -Message may scare people into clicking to find out how they can protect themselves *“I thought that was alarming that you could have it and not know and I’d want to click to find out what the symptoms were that I wouldn’t be aware of.” FP7, FG2* • **Evidence that the message may be ineffective but not harmful** -Message is true of all cancers and therefore does not communicate anything new *“That’s probably true of any type of cancer so I just thought you’re not telling me anything new.” MP7, FG3*
#3–9 out of 10 people can be cured when colon cancer is found early. (Perceived benefits)	Consider using this message • *N* = 45; 20 Men and 25 Women across all 6 focus groups • Wide range of ages • Ethnically diverse including Caucasian, Black, Asian and West Indian • Range of educational attainment	• **Evidence that the message may be effective** -Message grabs attention of audience members *“I thought [this message] was interesting.” FP, FG4* -Message is memorable *“I like [this message]. It’s simple. It’s easy for me to remember.” MP14, FG5* -Message has a positive, reassuring tone *“I think it’s optimistic. It makes you feel a little bit better. It tends to be more positive and hopeful. Better than the last one. This one makes you feel better.” FP10, FG2* -Message is credible *“As I said about my cousin being diagnosed; early detection saved her life, so this is the same thing. When you find things early it can save lives.” FP13, FG2* -Message is educational *“I never knew that [9 out of 10 people could be cured if cancer was found early].” MP15, FG5* -Message may prompt audience members to click to learn more and see their doctor *“[If I saw this message on my Facebook] I would go and read more about it and get information and try to talk to my family doctor.” FP12, FG2* • **Evidence that the message may be ineffective but not harmful** -Message is not scary enough to cause people to click on it *“I thought it was a good education piece. I don’t think I would have clicked on it. I just think that it’s really got to scare you and move you on the emotional level.” FP14, FG2* -Message will not grab attention on social media *“I think the idea is trying to get [people’s] attention and to try to get people to click on to it. With this statement, people will say ‘oh, yeah, that’s good.’ And then he or she will just go on to something else. They won’t click on it, in my opinion.” MP3, FG1*
#4 – Getting checked for colon cancer is easy and can be done in the privacy of your own home. (Perceived barriers)	Strongly consider using this message • *N* = 45; 20 Men and 25 Women across all 6 focus groups • Wide range of ages • Ethnically diverse including Caucasian, Black, Asian and West Indian • Range of educational attainment	• **Evidence that the message may be effective** -Message is credible *“[The message] mentions that getting checked for colon cancer is easy and can be done in the privacy of your own home. In fact, that’s a reality and that’s a real-life experience for me.” MP9, FG3* -Message has a positive tone *“I found [this message] the best. I find it to be very positive and it would encourage someone I believe to continue reading.” FP24, FG6* -Message is educational and comforting *“I like [this message] because I think a lot of people have the misconception that, all rubber glove and finger up the butt kind of thing at the doctor’s office. It’s comforting to know that you can do it yourself.” FP23, FG6* -Message addresses barriers to screening and may prompt audience members to take action *“The text that says you can do it easily, quickly and in privacy is really important. It’s kind of hard to do a sell on the purge before colonoscopy or having a colonoscopy so this gets people more, approachable for doing this and taking action.” MP7, FG3* • **No evidence that the message may be ineffective or harmful**
#7 – Colon cancer kills 9000+ Canadians every year. Don’t be one of them. (Perceived severity)	Proceed with caution • *N* = 45; 20 Men and 25 Women across all 6 focus groups • Wide range of ages • Ethnically diverse including Caucasian, Black, Asian and West Indian • Range of educational attainment	• **Evidence that the message may be effective** -Message grabs attention *“The “9000″ number caught my attention.” MP13, FG4* -Message is educational *“I had no idea it affected that many people. So that one really stood out.” FP21, FG5* -Message provides incentive to click and learn more *“The first part is kind of depressing and sad when you hear, 9000 died. But then, the second part gives you hope. Don’t be one of them so do something about it. It makes you want to take action, be assertive.” FP10, FG2* • **Evidence that the message may be ineffective but not harmful** -Message may be unclear *“I’m not really a numbers person. I can’t remember them or process them.” FP7, FG2* -Message is unappealing *“[This message] sounds less appealing to the reader.” MP9, FG3* -Message may not stand out among other ads on social media *“Well, if I were to be looking at my Facebook feed, I would ignore [this message] to be frank. They’re all trying to catch my attention and they do seem like other ads that I see.” MP21, FG6* • **Evidence that the message may be harmful** -Message tone is negative, discouraging, impersonal and fear mongering *“I agree, the scare tactics [of this message] … it’s too alarming and it’s not personal enough.” FP14, FG2* -Message may scare people and turn them away from screening *“I think for some people [this message] would put you in a depression. Some people may be scared and wouldn’t go for check-up. It may be very scary.” MP6, FG3*
#11 – Are you 50–74 years old? Healthcare providers recommend you get checked for colon cancer every 2 years. (Social norms and cues to action)	Consider using this message • *N* = 30; 17 Men and 13 Women across 4 focus groups • Wide range of ages but skewing younger • Mix of ethnicities but skewing Caucasian/White • Range of educational attainment but skewing towards lower educational attainment	• **Evidence that the message may be effective** -Message prompted participants to ask questions about protective measures *“Is [stool testing] every two years or can we request to do it every year?” MP10, FG3* -Message is clear and simple *“[This message] is really clear and descript.” MP9, FG3* -Message is educational *“I was reading [the message] about getting tested every two years. I didn’t know that. Every two years it said.” FP3, FG1* -Message references a trusted source *“[This message is my favorite because it is] bringing in a trusted authority like your health care provider.” MP7, FG3* • **Evidence that the message may be ineffective but not harmful** -Message is generic *“It just looks like every health pamphlet you’ve ever seen in a doctor’s office.” FP23, FG6* -Message doesn’t provide enough information about the screening test *“Which test are you talking about? Because I mean you don’t have colonoscopies every two years.” FP1, FG1*
#12 – Don’t flush it away. Test your poop for colon cancer today. It’s easy and can be done at home. (Perceived barriers and use of humour)	Proceed with caution • *N* = 45; 20 Men and 25 Women across all 6 focus groups • Wide range of ages • Ethnically diverse including Caucasian, Black, Asian and West Indian • Range of educational attainment	• **Evidence that the message may be effective** -Message grabs attention and is likely to stand out on social media *“If the purpose is to try to design an attention-getting campaign on social media then [this message] I think is effective.” MP13, FG4* -Audience appreciates the funny, playful tone *“It’s funny, I found it humorous. I thought it was for curiosity’s sake.” FP14, FG2* -Message is credible *“A friend of mine said she looked back, and she saw the blood and that’s how she found out that there was something wrong with her, [she had] colon cancer. She didn’t flush it. So you see, it happens.” FP13, FG2* -Message increases self-efficacy *“[This message] alleviates some of my concerns. It’s not the most comfortable thing to think about and this is sort of suggesting that “It’s easy and can be done at home.” And I feel less concerned about that.” MP21, FG6* -Message may prompt audience members to click to get more information about the test *“I would click on there to find out how easy it is. Then I would want to see how it works and makes me open to check for more information.” FP2, FG1* • **Evidence that the message may be ineffective but not harmful** -Audience may ignore the message *“[This message], I’d [scroll] right over that.” MP8, FG3* -Message isn’t as clever as it could be *“The [messages] that talk about poop specifically stand out to me. I don’t really care for them, but, I think they could have made it a little bit more clever.” FP23, FG6* • **Evidence that the message may be harmful** -Message is offensive *“Crap and poop and toilets and flushing. It disgusts me.” MP14, FG5*

Four messages (#1, #2, #3, #11) received a recommendation to consider using the message. These messages addressed perceived susceptibility, perceived benefits and social norms. These messages were seen as credible, positive or reassuring, simple and easy to understand; they also increased awareness about CRC risk and screening and prompted participants to ask questions or want to learn more about what they could do to lower their risk. Although these messages also attracted negative comments, these generally suggested the messages may be ineffective in some cases but not harmful.

Three messages (#1, #2 and #7) were designed to increase the perception of CRC risk in the audience. These messages elicited a range of reactions, including that the messages were scary enough to prompt people to take steps to protect their health, not scary enough, or too scary. One of the messages (#7) received a recommendation to proceed with caution because of the negative tone of the message and the fear triggered by it.

One message (#12) was designed to use humour to engage the audience. While some participants said the message caught their eye and appreciated the playful tone, others felt the message was offensive and unprofessional, leading to a recommendation of proceed with caution.

Focus group participants also discussed general considerations that may be useful when designing social media campaigns for cancer screening. Some participants distinguished between social and traditional mass media and said social media messages need to ‘stand out.’ Specifically, participants talked about the sheer amount of content and information that is competing for their attention on these platforms and that messages can easily get lost in this sea of information. Some participants look for ‘lighter content’ when using Facebook or other social media platforms and therefore they may actively avoid messages that sound like bad news. While some participants felt that Facebook was not a trusted source of health information, an important feature of the tested messages that conveyed credibility included the use of a Canadian domain (.ca). This suggested to the participants that the information was from a trusted source and not ‘just another ad trying to sell you something.’

## Discussion

Our study identified several social media messages that may be effective in encouraging CRC screening among Facebook users of screen-eligible age. Of the 7 messages with an overall recommendation, one was classified as strongly consider using this message, four as consider using this message and two as proceed with caution. Participants preferred social media messages that were believed to be credible, educational, and with a positive or reassuring tone. These messages tended to increase awareness about CRC risk and screening and prompted participants to ask questions and to want to learn more about what they could do to lower their risk. Messages that were viewed as humorous or offensive, or that had a negative or fearful tone were not well received by all participants and may garner mixed reactions. Using nationally specific websites to foster trust/credibility is suggested.

Our results are aligned with previous research exploring what types of messages may be most effective for CRC screening. For example, Kiviniemi et al. [22] found the greatest evidence for the behavioural change constructs of benefits, barriers, and perceived susceptibility, which underpin 5 of the 7 messages that our study is recommending for use in a future social media campaign. There are also similarities between our results and that of Weaver et al. [31] who developed text messages for CRC screening through focus groups with adults aged 50–75 years of age. Participants in their study identified the following characteristics for appealing text messages including having a positive or reassuring tone, and avoiding content that contains bad news or test results [31]. Although text messages may be inherently different from social media messages, some similarities likely remain given the bidirectional flow of information and the fact that social media accounts are also frequently accessed through mobile phones.

Our findings suggest that a fine balance is needed between instilling a sense of urgency through the use of fear-based messaging and instilling too much fear where the audience is put off by a message. Fear appeals including messages that address perceived susceptibility have been shown to be associated with screening outcomes such as intention or participation [22, 32]. This was also supported by our data where participants commented that certain messages were not ‘scary enough’ to prompt action (messages #1, #3). Participants in our study also described some messages as instilling a sense of urgency to act (message #2), while others felt that some messages may be ‘very scary’ and thus prevent people from taking action (message #7). The use of fear-based messaging requires further consideration by campaign planners given the mixed reactions including whether these could be addressed by audience segmentation.

Our data suggest that some people may be turning to Facebook for ‘lighter content’; as such, planners may wish to avoid any fear-based messaging. Interestingly, Carcioppolo et al. [33] explored the addition of humour to fear-based messages about colonoscopy screening and tested these online using Amazon’s Mechanical Turk platform. The authors found that messages with a mixed appeal (fear and humour combined) may be more effective at increasing screening intentions than fear-based messaging alone among those with high cancer worry [33]. This may suggest that the addition of humour may soften fear-based messaging that may otherwise scare the target audience. The one message that included humour in our study also had a mixed appeal with the perceived barriers construct and reactions to this message led to a recommendation of proceed with caution. Additionally, Carcioppolo et al. [33] identified opportunities for audience segmentation including among those with high cancer worry which may be more receptive to mixed appeal messages. Further exploration regarding which segment of social media users would engage with mixed appeal messaging is needed.

The results of our study must be considered in light of the study’s strengths and limitations. Due to the limited time available in each focus group, we were not able to test all messages in all focus groups. This resulted in some messages not garnering sufficient comments to make a recommendation. However, we were still able to make an informed recommendation on 7 of the tested messages. Our study was rigorously conducted as analysis was completed independently by two coders and supported by extensive discussion within the broader study team. Given that participants in our study were only from Ontario, our findings may not be generalizable to other populations. However, our study utilized random digit dialing (inclusive of both landlines and cellphones) for recruitment and to ensure variation among participant demographics. While we did not limit inclusion criteria to those at average risk of disease, the messages in our study were targeted to those at average risk of disease and some consideration to this and the potential for audience segmentation must be given in future work (e.g. age, gender, prior screening history). It is also important to note that our study focused on one social media platform. As such, our findings may not be transferable to other platforms. Despite this, our study fills an important gap in the literature as it identifies potential messages that program planners may use to maximize screening efforts and characteristics of effective social media messages for CRC screening specifically.

## Conclusions

Our study provides insights into how behaviour change theory and focus group input can be used to develop social media messages for cancer screening. Our study has identified social media messages that may be preferred by individuals of screen-eligible age. These messages, coupled with appropriate images or design can be used in a randomized controlled trial on Facebook to evaluate effectiveness of social media messaging to increase CRC screening uptake. Further research could also extend this work to explore whether the messages would need to be refined to address the needs of those at increased risk of disease and whether audience segmentation could be used for messages with mixed reactions.

## Supplementary Information

**Additional file 1.** List of messages tested in focus groups.

## Data Availability

The dataset used and analysed during this study are available from the corresponding author on reasonable request.

## Abbreviations

COVID-19: Coronavirus disease 2019
CRC: Colorectal cancer
SRQR: Standards for Reporting Qualitative Research
