# Supplementary material for: Using Facebook to promote the uptake of colorectal cancer screening
Source: BMC Public Health. 2022 Feb 15;22:323. doi: 10.1186/s12889-022-12732-w (PMC8848656; doi:10.1186/s12889-022-12732-w)
Supplement: Supplementary file 1 — Additional file 1. List of messages tested in focus groups. [file 12889_2022_12732_MOESM1_ESM.pdf]

Additional File 1. List of messages tested in focus groups.

The messages are presented in the following format as per Facebook ad layout

# --- Message #
50 or older? You may be at risk for colon cancer. --- Primary headline
<IMAGE TBC> --- Image placeholder
GETCHECKEDTODAY.CA --- Website link
Get checked today --- Headline
LEARN MORE --- Call to action button

**#1**<br>Did you know your risk of colon cancer increases after 50?<br><IMAGE TBC><br>GETCHECKEDTODAY.CA<br>**Get checked today**<br>**LEARN MORE**	**#10**<br>Because taking care of your health is never embarrassing.<br><IMAGE TBC><br>GETCHECKEDTODAY.CA<br>**Get checked for colon cancer today**<br>**LEARN MORE**
**#2**<br>You could have colon cancer right now—and have no clue.<br><IMAGE TBC><br>GETCHECKEDTODAY.CA<br>**Now's the time to get tested**<br>**LEARN MORE**	**#11**<br>Are you 50-74 years old? Healthcare providers recommend you get checked for colon cancer every 2 years.<br><IMAGE TBC><br>GETCHECKEDTODAY.CA<br>**Get checked for colon cancer today**<br>**LEARN MORE**
**#3**<br>9 out of 10 people can be cured when colon cancer is found early.<br><IMAGE TBC><br>GETCHECKEDTODAY.CA<br>**50 to 74? Get checked today**<br>**LEARN MORE**	**#12**<br>Don't flush it away. Test your poop for colon cancer today. It's easy and can be done at home.<br><IMAGE TBC><br>GETCHECKEDTODAY.CA<br>**Get checked today**<br>**LEARN MORE**
**#4**<br>Getting checked for colon cancer is easy and can be done in the privacy of your own home.<br><IMAGE TBC><br>GETCHECKEDTODAY.CA<br>**Get checked today**<br>**LEARN MORE**	**#14**<br>Because living with half a colon isn't easy.<br><IMAGE TBC><br>GETCHECKEDTODAY.CA<br>**50 to 74? Get tested for colon cancer**<br>**LEARN MORE**

**#5**<br>Think you don't need to get checked for colon cancer because no one in your family has had it? Think again.<br><IMAGE TBC><br>GETCHECKEDTODAY.CA<br>**Talk to your healthcare provider today**<br>**LEARN MORE**	**#17**<br>Everyone passes poop. Now, let's see if your poop passes the test.<br><IMAGE TBC><br>GETCHECKEDTODAY.CA<br>**Get checked for colon cancer today**<br>**LEARN MORE**
**#6**<br>"Checking for colon cancer was easy, quick and private."— <First Name><br><Image featuring a headshot of a patient/the source of quote above><br>GETCHECKEDTODAY.CA<br>**Get checked today**<br>**LEARN MORE**	**#21**<br>If you're 50-74, it's time to get your rear in gear.<br><IMAGE TBC><br>GETCHECKEDTODAY.CA<br>**Get checked for colon cancer today**<br>**LEARN MORE**
**#7**<br>Colon cancer kills 9,000+ Canadians every year. Don't be one of them.<br><IMAGE TBC><br>GETCHECKEDTODAY.CA<br>**This quick test can protect your health**<br>**LEARN MORE**	**#22**<br>Think you don't have the time to get tested? Treating colon cancer takes even longer.<br><IMAGE TBC><br>GETCHECKEDTODAY.CA<br>**Get checked for colon cancer today**<br>**LEARN MORE**
**#8**<br>You want to be there for all the big moments. Make sure colon cancer doesn't get in the way.<br><Image of a family><br>GETCHECKEDTODAY.CA<br>**50 to 74? Get checked today**<br>**LEARN MORE**	**#23**<br>Colon cancer can take years before it grows enough to make you sick. But why wait that long?<br><IMAGE TBC><br>GETCHECKEDTODAY.CA<br>**Get checked for colon cancer today**<br>**LEARN MORE**
**#9**<br>Protect your health by getting checked for colon cancer.<br><IMAGE TBC><br>GETCHECKEDTODAY.CA<br>**Find out about home testing**<br>**LEARN MORE**	**#24**<br>"I'm so grateful that I took the time to get tested for colon cancer. Those few minutes saved my life."— <First Name><br><Image featuring a headshot of a patient/the source of quote above><br>GETCHECKEDTODAY.CA<br>**Get checked today**<br>**LEARN MORE**
